# Progranulin Mutation Manifesting as Parkinson Disease: A Case Series from the PADUA‐CESNE Cohort

**DOI:** 10.1002/mdc3.70064

**Published:** 2025-04-04

**Authors:** Giulia Bonato, Marta Campagnolo, Aron Emmi, Valentina Misenti, Tommaso Carrer, Carmelo Fogliano, Leonardo Salviati, Miryam Carecchio, Angelo Antonini

**Affiliations:** ^1^ Parkinson and Movement Disorders Unit, Center for Rare Neurological Diseases (ERN‐RND), Department of Neuroscience University of Padova Padova Italy; ^2^ Center for Neurodegenerative Disease Research (CESNE) University of Padova Padova Italy; ^3^ Padova Neuroscience Center (PNC) University of Padova Padova Italy; ^4^ Department of Neuroscience, Neurology University of Padova Padova Italy; ^5^ Institute of Human Anatomy University of Padova Padova Italy; ^6^ Clinical Genetics Unit, Department of Women and Children's Health University of Padova Padova Italy

**Keywords:** genetics, parkinsonism, progranulin, skin biopsy, synuclein

## Abstract

**Background:**

Mutations in progranulin (*GRN*) are associated with frontotemporal dementia, although a Parkinson disease (PD) phenotype is uncommon, especially in young patients.

**Cases:**

We report three subjects from the PADUA‐CESNE cohort, meeting diagnostic criteria for PD, with onset under age 55. All had good response to dopaminergic therapy, abnormal dopamine transporter single‐photon emission computed tomography striatal uptake and a disease course consistent with PD, without clear atypical features, behavioral, or cognitive deficits. Genetic testing (next‐generation sequencing [NGS] panel) revealed three different variants in *GRN* gene. Skin biopsy immunohistochemistry analysis showed phosphorylated α‐synuclein deposition in two and was negative in one subject.

**Conclusions:**

Our findings expand the phenotypic spectrum of *GRN* mutations, showing that patients can present with clinical manifestations of PD, including phosphorylated synuclein pathology in the skin, with a relatively young age of onset. Our observations support the use of broad‐spectrum NGS panels to properly guide patients in counseling and accurately allocate them to disease‐modifying therapies.

*GRN* gene mutations have been associated to a wide spectrum of clinical phenotypes, mainly behavioral variant frontotemporal dementia (FTD), with typical TAR DNA‐binding protein 43 (TDP‐43) pathology, but also primary progressive aphasia, atypical Alzheimer's disease, and atypical parkinsonism, mainly corticobasal syndrome (CBS).^1^ Extrapyramidal signs are also frequently observed in *GRN*‐related FTD, usually occurring after the onset of behavioral or cognitive symptoms in the fifth to sixth decade, in association with apraxia and dystonia, featuring a CBS phenotype.^2^ *GRN* mutations manifesting as Parkinson's disease (PD), with typical rest tremor, rigidity, bradykinesia and asymmetric symptom onset, without cognitive involvement, are very uncommon,^3^, ^4^ especially in early onset phenotypes.^5^

We report three subjects who received a diagnosis of PD with symptoms onset under 55 years of age, all carrying mutations in *GRN* gene. We describe clinical and cognitive features, disease course, and results from immunohistochemistry analysis on skin biopsy using antibodies against phosphorylated synuclein (pSyn) and tau (pTau). Subjects are part of our PADUA‐ CESNE cohort undergoing biological, genetic, and clinical characterization of PD at the Movement Disorders Unit of the University of Padova, according to previously published protocols.^5^, ^6^

## Case Series

### Case 1

This right‐handed 42‐year‐old patient complained of difficulty in writing at 40 years of age, followed by rest tremor and slowness in the right upper limb. He also reported constipation, mild hyposmia, and vivid dreams consistent with rapid eye movement (REM) behavior disorder (RBD). His mother died from malignant brain tumor, but possible parkinsonism was also reported; his maternal grandfather died at age 68 with a diagnosis of PD starting in the fifth decade, later complicated by cognitive decline; and his paternal grandmother had dementia with behavioral problems from the fifth decade and died in the sixth decade.

Clinical examination showed hypomimia, hypophonia, normal walking speed with a tendency to drag his right leg, absent arm swing on the right side, marked rest tremor on the right hand with postural and kinetic component, marked rigidity of the right limbs, severe bradykinesia of the right upper limb, and moderate slowness on the left hand and right foot. Cranial nerve examination was unremarkable. The Movement Disorders Society Modified Unified Parkinson's Disease Rating Scale (MDS‐UPDRS) III score was 30. Brain magnetic resonance imaging (MRI) was unremarkable; dopamine transporter single‐photon emission computed tomography (DAT‐SPECT) showed reduced tracer uptake in left putamen, with moderate decrease in left caudate nucleus and right posterior putamen binding. The patient was diagnosed with PD^5^ and dopaminergic therapy was started (levodopa/carbidopa 300 mg/d, selegiline 5 mg/d) with a good and sustained response on both tremor and bradykinesia. Peak‐dose dyskinesias, mild motor fluctuations, and orthostatic hypotension after therapy intake developed 1 year later, therefore, the therapy was adjusted accordingly and fragmented in multiple low‐dose administrations with good results. Five years after disease onset there is sustained response to levodopa (LD) therapy, without atypical features (Video 1), and cognitive evaluation is normal (Mini‐Mental State Examination [MMSE]: 28/30, Montreal Cognitive Assessment [MoCA]: 27/30; slight difficulties in sustained concentration and clock drawing test, absence of frontal, or behavioral signs).

**Video 1:** Neurological examination of case 1 after 5 years of follow‐up, during on‐state. The patient can walk at a brisk pace, without falls or difficulties in turning or maintaining his stance; arm swings are reduced on his left side, whereas there is dystonic posture on his right arm and dyskinesias of the head and rarely of lower limbs. There is bradykinesia at finger tapping and hand movements more prominent on his right side, with involvement of his left limbs too; rigidity can be detected on his right upper limb. There is no prominent kinetic tremor, but there is mild postural tremor on his right hand and mild rest tremor (prevalent on the right side) while he is standing upright. The patient's voice is hypophonic, without dysarthria; cranial nerves' examination is normal, eye movement range is preserved.

**FIG. 1:**
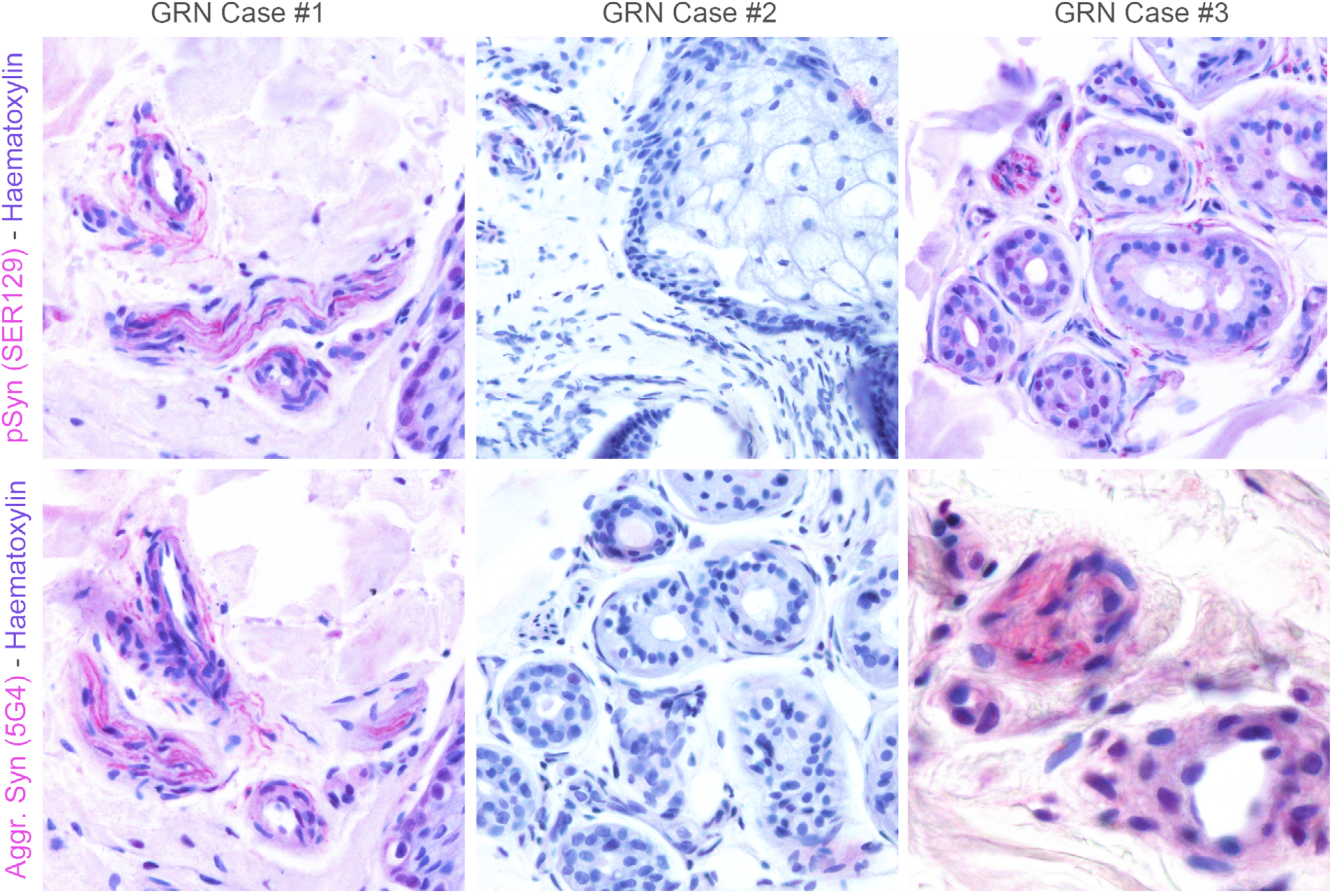
α‐Synuclein deposition (pSyn SER129 and 5G4) in peripheral nerve fibers detected via immunoperoxidase in punch‐skin biopsies of *GRN* mutation carriers (20× magnification). Patients 1 and 3 display clear positivity for α‐synuclein in small nerve fibers innervating vascular and glandular structures of the derma. Patient 2 was negative for pathological α‐synuclein immunohistochemistry.

Genetic testing documented a single pathogenic variant in *GRN* gene (NM_002087.2:c.813_816del, p.[Thr272Serfs*10], American College of Medical Genetics and Genomics [ACMG] 5)^5^ recurrent in literature (Table 1). Skin biopsy histopathology revealed the presence of pSyn and aggregated α‐synuclein (5G4), without pTau deposition (Fig. 1).

**TABLE 1:** Genetic findings in the subjects and ACMG interpretation

	Case 1	Case 2	Case 3
*GRN* variant	NM002087.2:c.813_816del p.(Thr272Serfs*10) Heterozygous	NM002087.2:c.1633C>T p.(Pro545Ser) Heterozygous	NM002087.2:c.415 T>C p.(Cys139Arg) Heterozygous
ACMG class	5	3	4
ACMG items applied	PS4, PVS1, PM2, PS3, PP5	PM2, PP3, PS3	PM2, PP3, PP5, PS3
MAF	0	0	0.000179
Conservation PhyloP100	1.4	4.7	6.4
In silico prediction tools:	/		
Sift		0	0
PolyPhen‐2		1	1
CADD		29.2	24.2
Aggregated score FranklinGenoox ClinVar	Pathogenic	0.83 (pathogenic moderate) /	0.85 (pathogenic moderate) uncertain/conflicting data
Reported in literature	Yes (PGRN levels reduction, PMID 18768919)	No	Yes (PGRN levels reduction, PMID 23724906)
Segregation analysis	Not available	Not available	Not available

Abbreviations: ACMG, American College of Medical Genetics and Genomics; MAF, Minor Allele Frequency; PGRN, progranulin protein; PMID, PubMed identifier.

### Case 2

This 54‐year‐old woman was diagnosed with PD presenting at age 53 with bradykinesia in the left limbs (MDS‐UPDRS III score: 6). She also reported constipation and a sleep disorder consistent with RBD. Her family history and previous medical history were unremarkable. DAT‐SPECT showed tracer uptake defect in both putamina, with prevalence on the right side. Ropinirole 4 mg and levodopa 100 mg bid were started with good results (MDS‐UPDRS III score: 2, MDS‐UPDRS IV score: 5). Neuropsychological assessment revealed an overall normal cognitive status (MMSE: 26.2/30, MoCA: 26.52/30) with no evidence of frontal or behavioral deficits.

Genetic testing showed a heterozygous *GRN* variant (NM_002087.2:c.1633C>T, p.(Pro545Ser), ACMG 3). The variant has Minor Allele Frequency (MAF) 0 and affects a residue located in domain E that is highly conserved in vertebrate species and among the other progranulin protein (PGRN) structural domains (Fig. S1A,B). The imino‐acid proline545 seemed to have a relevant role in the three‐dimensional (3D) structure of the protein domain at in silico modeling (Fig. S1C,D), stabilizing β‐sheet structure through interaction with glutamine531 and maintaining the correct angle between the end of the β‐sheet and the subsequent loop through its limited rotation ability. Substitution with serine could alter this angle and the overall 3D conformation of the protein domain. These data and prediction software both supported a possible deleterious effect (Table 1).

At the 6‐month follow‐up visit, the patient reported subjective difficulty in swallowing, not confirmed at objective tests, without additional symptoms. Cardiac (iodine‐123 meta‐iodobenzylguanidine) 123I‐MIBG scintigraphy found a normal heart sympathetic innervation.

Skin biopsy was negative for pSyn as well as for pTau aggregates (Fig. 1).

### Case 3

This 31‐year‐old man came to our attention because of slowness and a decrease in dexterity in his right upper limb worsening over a 2‐year timeframe. His family history was unremarkable. DAT‐SPECT documented reduced tracer uptake bilaterally in the striatum, and brain MRI was unremarkable, in line with diagnosis of PD. In the following year, he was treated with levodopa 400 mg/d, rasagiline 1 mg/d, and pramipexole 1.05 mg/d with benefit on motor symptoms. Cognitive functions were normal on formal neuropsychological assessment (MMSE: 30/30, MoCA: 29/30), without frontal or behavioral involvement.

Genetic testing showed a previously reported heterozygous *GRN* variant (NM_002087.2:c.415 T > C, p.[Cys139Arg], ACMG 4, Table 1). Skin biopsy was positive for aggregated α‐synuclein and pSyn deposition without pTau deposition (Fig. 1).

## Discussion

Extrapyramidal signs are reported in up to 87% of cases in *GRN* mutations carriers, often as a part of the FTD or CBS phenotypic spectrum.^2^ Our three cases presented with clinical onset, disease course, and response to dopaminergic therapy consistent with PD. Most importantly there were no features consistent with the diagnosis of FTD or PD‐dementia.

In the last decades, a limited number of patients carrying *GRN* mutations presenting with clinical features consistent with diagnosis of PD have been described.^3^, ^4^, ^7^ These patients' first clinical presentation was usually after their fifth decade, with asymmetric rest tremor, akinetic‐rigid syndrome, and good response to dopaminergic therapy. However, in all cases atypical features appeared either at onset or after few years, such as falls, myoclonus, pyramidal signs, apraxia, rapidly progressive cognitive, and behavioral disturbances consistent with FTD or leading to a later diagnosis of atypical parkinsonism, CBS, or PD‐dementia, with poor prognosis.^3^ Except for one patient, all the cases reported so far had a positive family history for dementia or parkinsonism.^3^, ^4^

In our cohort, *GRN* mutation carriers showed features indistinguishable from PD, and early age at onset, with two patients presenting motor onset before 40 years of age (case 1 and 3), an age range usually more likely to be associated with mutations in recessive PD genes such as *PRKN*, *PINK1*, or *DJ1*.^5^ Moreover, in case 2 and 3 family history was unremarkable for neurological conditions, which we believe enforces the importance of performing comprehensive genetic tests to younger patients irrespective of a positive family history.

In recent years, skin biopsy has become a useful tool in documenting presence of pathological aggregates in synucleinopathies.^6^ In case 1 and 3, we found pathological pSyn deposition, whereas in case 2 there were no synuclein peripheral aggregates. This is the first study reporting immunohistochemistry analysis of skin biopsies of *GRN* mutation carriers, and our findings highlight the presence of pSyn with a pattern similar to non‐genetic PD also in this rare genetic mimic.

In line with our findings, previous neuropathological studies on brain tissue of *GRN* mutation carriers also showed mixed results. Although in the majority of cases ubiquitin inclusion and TDP43 pathology were the only finding regardless of clinical phenotype, some studies documented the coexistence of α‐synuclein deposits along with tau and TDP43 pathology.^7^ Moreover, recent evidence has shown a possible facilitating effect of progranulin dysfunction on α‐synuclein deposition through impairment of Prosaposin processing and glucocerebrosidase activity as well as involvement of the autophagy‐lysosome pathway, a recurring disease mechanism for neurodegenerative diseases.^7^, ^8^ Low plasma progranulin levels, once used to predict an underlying *GRN* mutation in the pre‐next generation sequencing era and its biological effect, have been demonstrated in a wide range of neurodegenerative diseases, postulating a role of PGRN as a risk factor for neurodegeneration.^9^

Specific *GRN* variants' effects, especially for missense variants, may also play a role in different phenotypes and histological findings. The absence of synuclein deposition in case 2 could also be explained by a milder phenotype or by brain‐first disease, as hinted by normal cardiac 123I‐MIBG scintigraphy.^10^ Unfortunately, we were not able to measure PGRN plasma levels in these patients and use them for correlation with other biological and clinical data.

In conclusion, our findings confirm the phenotypic variability in *GRN* mutation carriers, which can also manifest with PD symptoms at an early age, further emphasizing the importance of performing a thorough genetic analysis in patients with parkinsonism. This is also crucial for future treatment. Trials for disease modifying therapies in *GRN* mutation carriers are now ongoing and may consider inclusion of other phenotypic manifestations beside FTD. Moreover, our results encourage the use of accurate genetic screening in PD disease modifying studies to exclude subject with other genetically determined pathology.

## Author Roles

(1) Research project: A. Conception, B. Organization, C. Execution; (2) Data Analysis: A. Design, B. Execution, C. Review and Critique; (3) Manuscript Preparation: A. Writing of the first draft, B. Review and Critique.

G.B.: 1A, 1B, 1C, 2B, 3A

M.C.: 1A, 1C, 2C, 3B

A.E.: 1A, 1C, 2B, 3A, 3B

T.C.: 1C

CF: 1C

F.P.: 1C

L.S.: 1B, 1C, 2B, 3B

M.C.: 1A, 1C, 2C, 3B

A.A.: 1A, 1B, 1C, 2C, 3B

## Disclosures

**Ethical Compliance Statement:** The work was conducted in compliance of the Padova University Hospital ethical guidelines and local Ethical committee, as part of a biocollection program for neurodegenerative disorders. Written informed consent was obtained from patients for all study procedures. We confirm that we have read the Journal's position on issues involved in ethical publication and affirm that this work is consistent with those guidelines.

**Funding Sources and Conflict of Interest:** No specific funding was received for this work. The authors declare that there are no conflicts of interest relevant to this work.

**Financial Disclosures for the Previous 12 Months:** The authors declare no relevant financial interests that relate to the research described in this paper.

## Supporting information


**Figure S1.** Proline545 conservation analysis and protein three‐dimensional structure of domain E, affected by the p.(Pro545Ser) variant. The 3D structure of the PGRN domain was modeled on the published structure of human progranulin domain A (PMID 18359860, PDB code 2JYE) as reported (PMID 31997039). (**A**) Alignments of progranulin domain E from different vertebrate species. The arrowhead indicates proline545. (**B**) Alignments of the seven granulin domains of human progranulin. The arrowhead indicates proline545. (**C**) Interaction between proline545 and glutamine531, that seems to play a role in stabilizing beta sheet 3D structure. (**D**) The arrowhead indicates the angle between proline 545 at the end of the beta‐sheet and the subsequent loop, that seems to be stabilized by proline545, as a rigid imino‐acid with limited rotation ability within the polypeptide chain. Although a strict application of ACMG criteria does not allow to classify the variant in class 4 or 5, classifying as ACMG class 3, these data and prediction tools suggest a possible deleterious role for this substitution in Case 2.

## Data Availability

The data that support the findings of this study are available from the corresponding author upon reasonable request.
