# Supplementary material for: Progranulin Mutation Manifesting as Parkinson Disease: A Case Series from the PADUA‐CESNE Cohort
Source: Mov Disord Clin Pract. 2025 Apr 4;12(7):998–1002. doi: 10.1002/mdc3.70064 (PMC12275000; doi:10.1002/mdc3.70064)
Supplement: Supplementary file 1 — Figure S1. Proline545 conservation analysis and protein three‐dimensional structure of domain E, affected by the p.(Pro545Ser) variant. The 3D structure of the PGRN domain was modeled on the published structure of human progranulin domain A (PMID 18359860, PDB code 2JYE) as reported (PMID 31997039). (A) Alignments of progranulin domain E from different vertebrate species. The arrowhead indicates proline545. (B) Alignments of the seven granulin domains of human progranulin. The arrowhead indicates proline545. (C) Interaction between proline545 and glutamine531, that seems to play a role in stabilizing beta sheet 3D structure. (D) The arrowhead indicates the angle between proline 545 at the end of the beta‐sheet and the subsequent loop, that seems to be stabilized by proline545, as a rigid imino‐acid with limited rotation ability within the polypeptide chain. Although a strict application of ACMG criteria does not allow to classify the variant in class 4 or 5, classifying as ACMG class 3, these data and prediction tools suggest a possible deleterious role for this substitution in Case 2. [file MDC3-12-998-s001.docx]

**Progranulin mutation manifesting as Parkinson disease: a case series from the PADUA- CESNE cohort**

**Movement Disorders Clinical Practice**

Giulia Bonato, MD ^1,2,3,4^; Marta Campagnolo, MD PhD ^1,2,4^; Aron Emmi, PhD ^2,5^; Valentina Misenti, PhD ^1,2,4^; Tommaso Carrer, MD ^1,4^; Carmelo Fogliano, MD ^1,4^; Leonardo Salviati, MD PhD ^6^; Miryam Carecchio, MD PhD ^1,2,3,4^; Angelo Antonini, MD PhD ^1,2,3,4^

*^1^ Parkinson and Movement Disorders Unit, Center for Rare Neurological Diseases (ERN-RND), Department of Neuroscience, University of Padova, 35128 Padova, Italy*

*^2^ Center for Neurodegenerative Disease Research (CESNE), University of Padova, 35128 Padova, Italy*

*^3^ Padova Neuroscience Center (PNC), University of Padova, 35128 Padova, Italy*

*^4^ Department of Neuroscience, Neurology, University of Padova, 35128 Padova, Italy*

*^5^ Institute of Human Anatomy, University of Padova, 35128 Padova, Italy*

*^6^ Clinical Genetics Unit, Department of Women and Children’s health, University of Padova, 35128 Padova, Italy*

***Corresponding author***

Prof. Angelo Antonini

Email: [angelo.antonini@unipd.it](mailto:angelo.antonini@unipd.it)

**Supplementary Material**


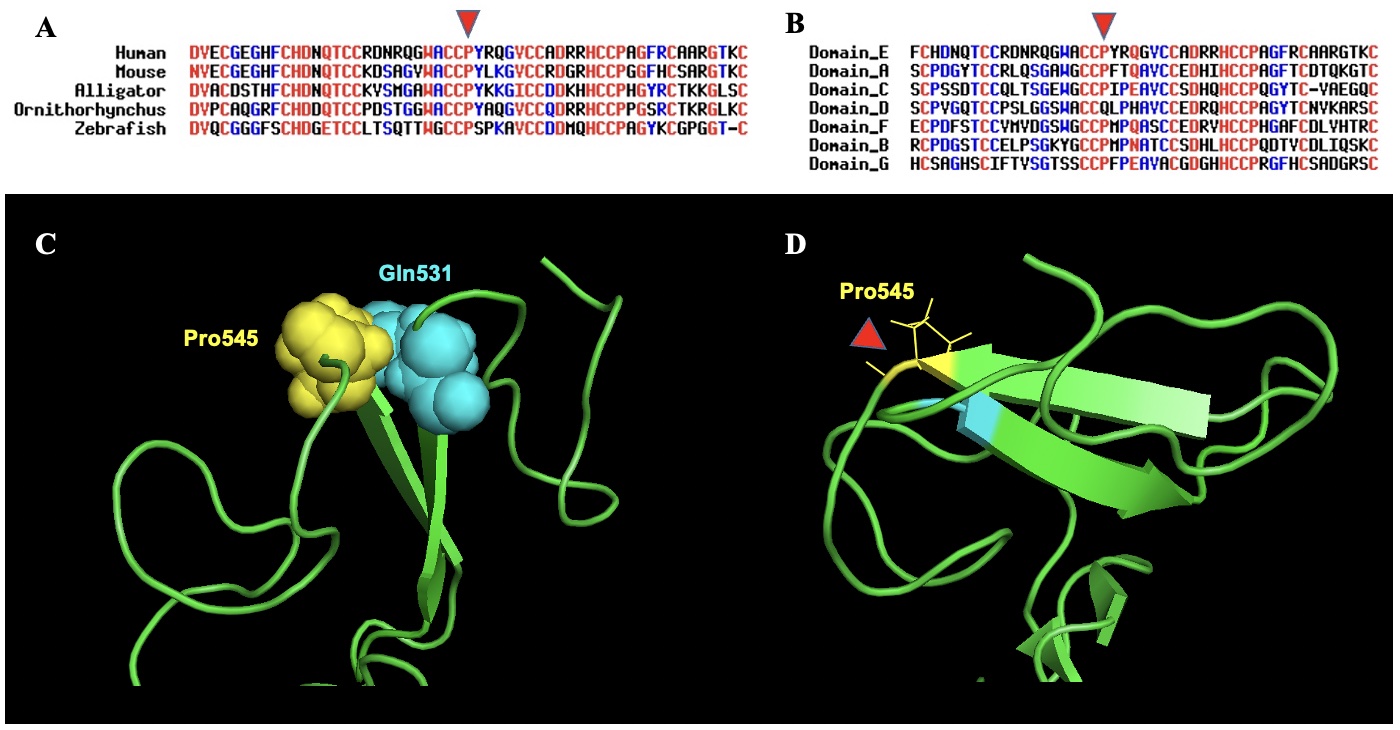


**Supplementary Figure S1.** Proline545 conservation analysis and protein three-dimensional structure of domain E, affected by the p.(Pro545Ser) variant. The 3D structure of the PGRN domain was modeled on the published structure of human progranulin domain A (PMID 18359860**,** PDB code 2JYE) as reported (PMID 31997039). **A)** Alignments of progranulin domain E from different vertebrate species. The arrowhead indicates proline545. **B)** Alignments of the seven granulin domains of human progranulin. The arrowhead indicates proline545. **C)** Interaction between proline545 and glutamine531, that seems to play a role in stabilizing beta sheet 3D structure. **D)** The arrowhead indicates the angle between proline 545 at the end of the beta-sheet and the subsequent loop, that seems to be stabilized by proline545, as a rigid imino-acid with limited rotation ability within the polypeptide chain. Although a strict application of ACMG criteria does not allow to classify the variant in class 4 or 5, classifying as ACMG class 3, these data and prediction tools suggest a possible deleterious role for this substitution in Case 2.

**Supplementary Video 1**. Neurological examination of Case 1 after 5 years of follow-up, during on-state. The patient can walk at a brisk pace, without falls or difficulties in turning or maintaining his stance; arm swings are reduced on his left side whereas there is dystonic posture on his right arm and dyskinesias of the head and rarely of lower limbs. There is bradykinesia at finger tapping and hand movements more prominent on his right side, with involvement of his left limbs too; rigidity can be detected on his right upper limb. There is no prominent kinetic tremor, but there is mild postural tremor on his right hand and mild rest tremor (prevalent on the right side) while he is standing upright. The patient’s voice is hypophonic, without dysarthria; cranial nerves’ examination is normal, eye movement range is preserved.
